# Supplementary figures and images for: CD169-mediated restrictive SARS-CoV-2 infection of macrophages induces pro-inflammatory responses
Source: PLoS Pathog. 2022 Oct 24;18(10):e1010479. doi: 10.1371/journal.ppat.1010479 (PMC9632919; doi:10.1371/journal.ppat.1010479)

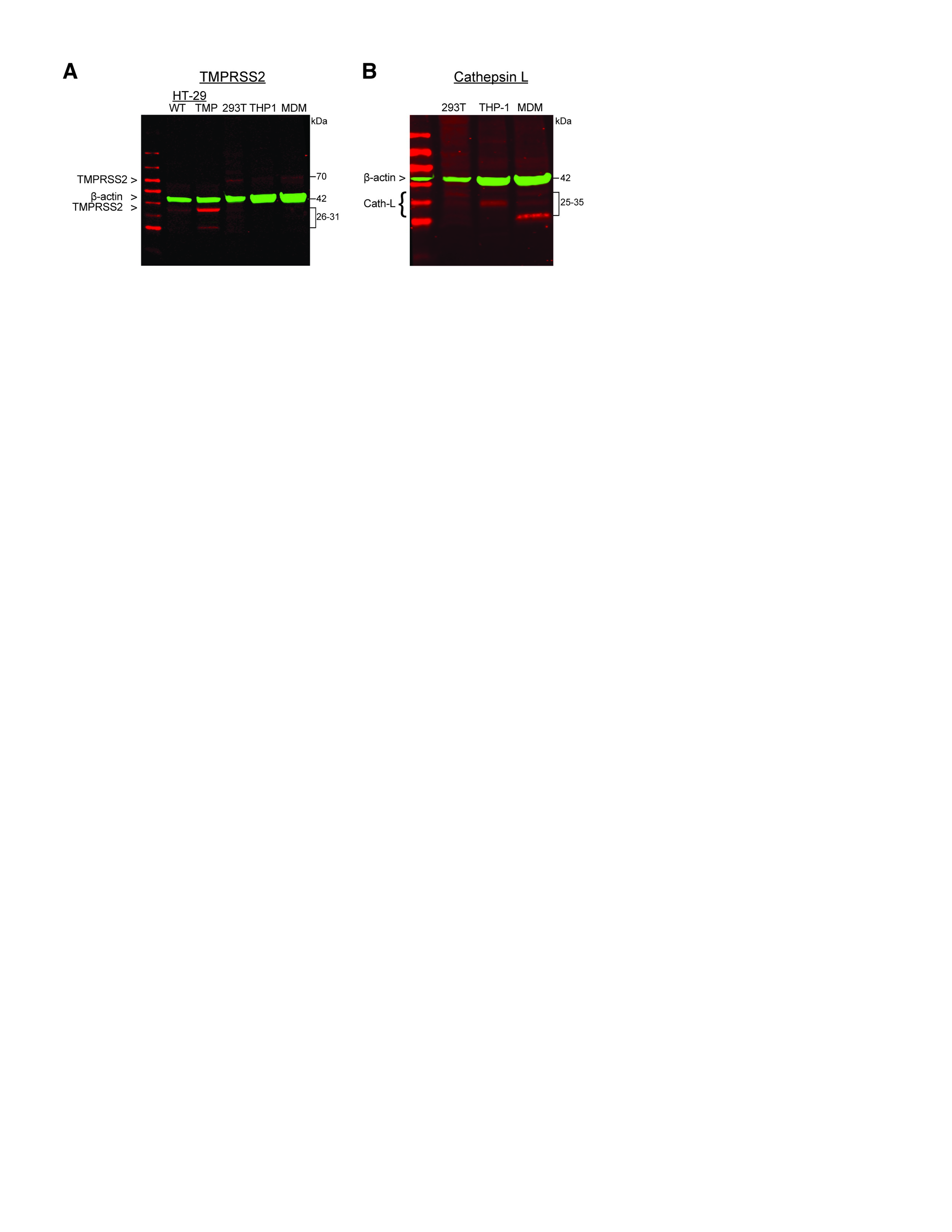

Supplement: S1 Fig — (A-B) Western blot analysis for TMPRSS2, uncleaved form detected in HEK293T and macrophages (A) and the different isoforms of Cathepsin-L, detected in in THP1 and macrophages (B) expression in wildtype and transduced HT-29 cells (control), HEK293T, THP1/PMA macrophages, and primary MDMs from multiple donors. β-actin was probed as a loading control. (TIF) [file ppat.1010479.s001.tif]

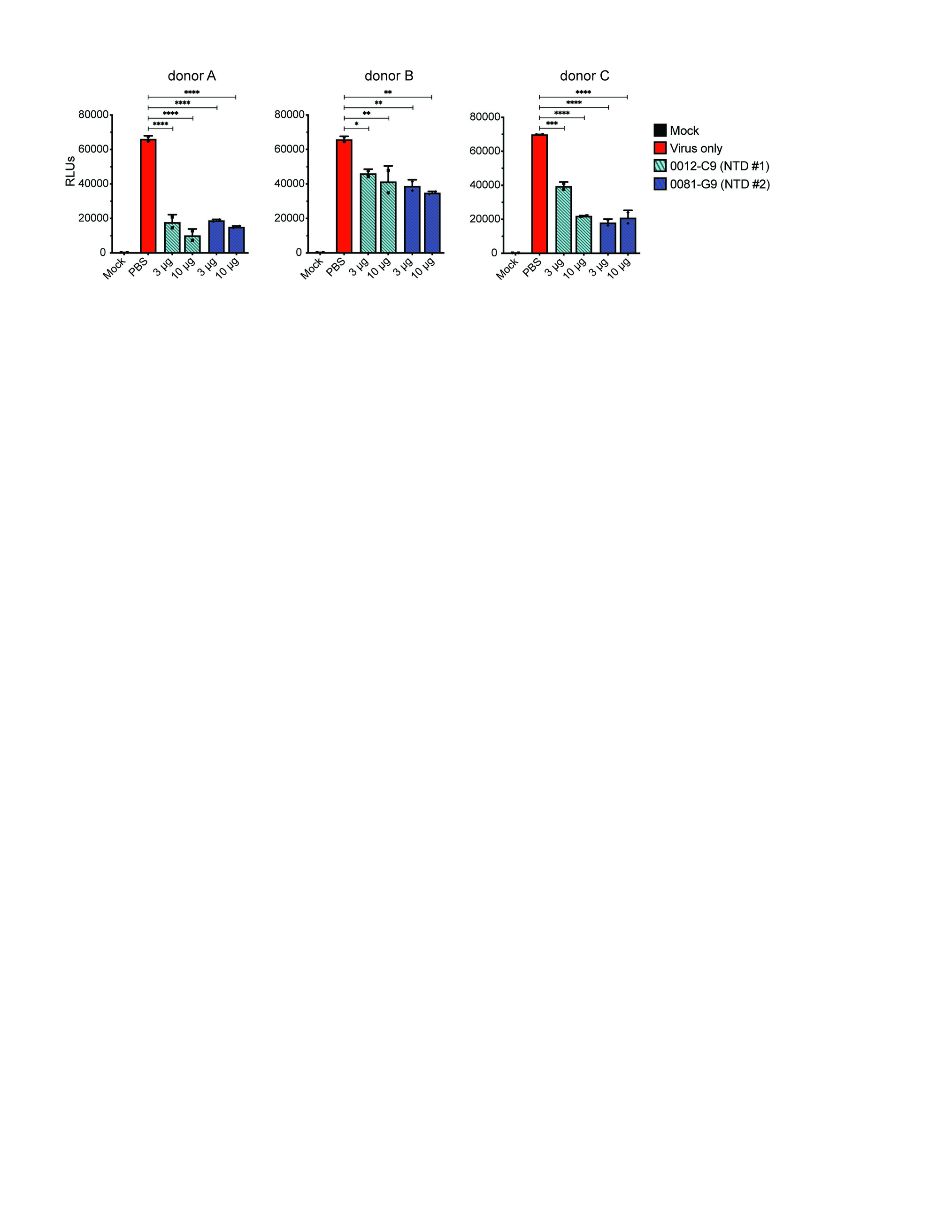

Supplement: S2 Fig — SARS-CoV-2 S-pseudotyped lentivirus (20 ng based on p24Gag) was pre-incubated with indicated anti-Spike neutralizing antibodies for 30 mins at 37°C, followed by infection of primary MDMs for 3 days. Relative infection quantified by luciferase activity from whole cell lysates. Data are representative of 2 independent experiments, from 3 different donors each. Mock: no virus added, PBS: no pre-incubation of virus with antibody. The means ± SEM are shown. P-values: one-way ANOVA followed by the Dunnett’s post-test comparing to untreated (PBS) control, *: p < 0.05; **: p < 0.01; ***: p < 0.001; ****: p < 0.0001. (TIF) [file ppat.1010479.s002.tif]

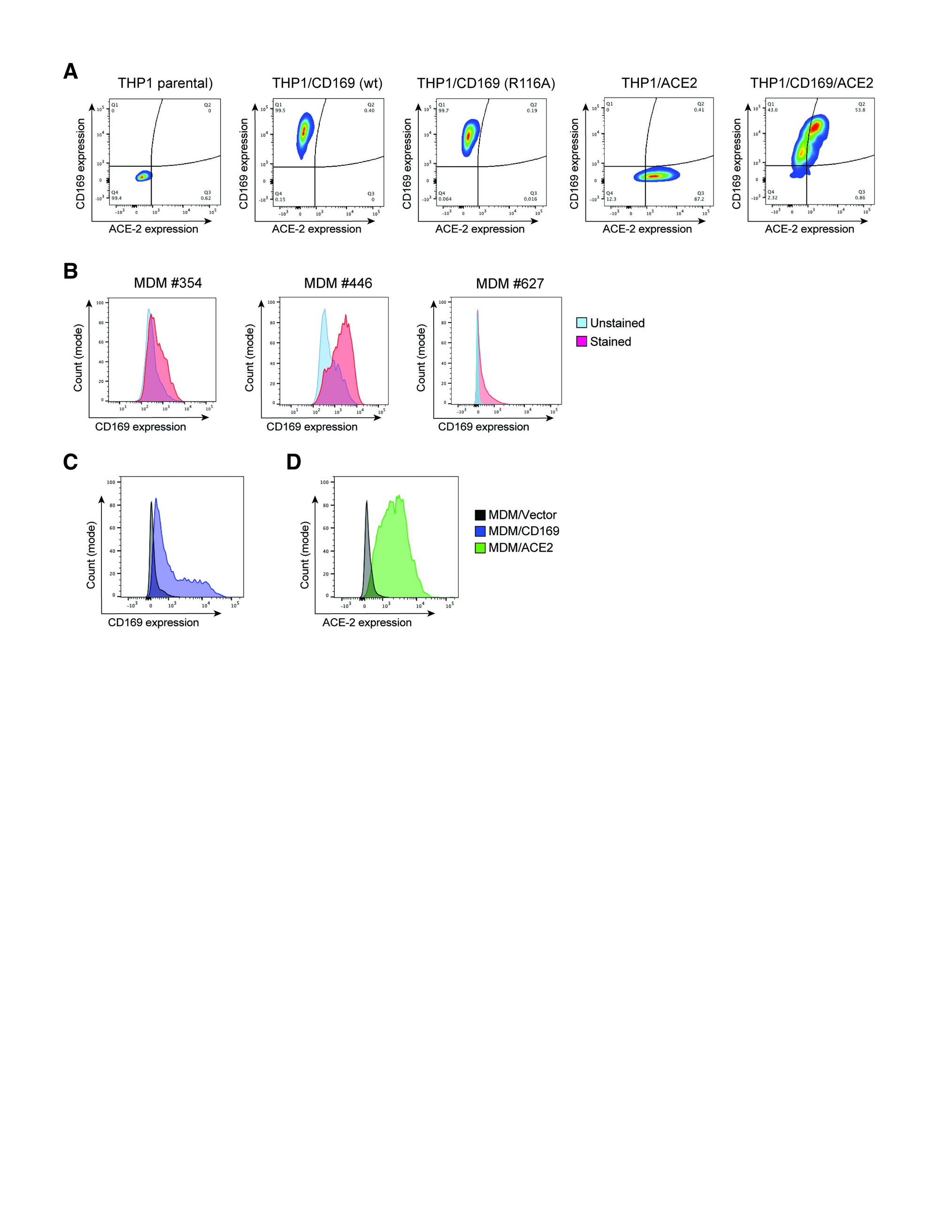

Supplement: S3 Fig — (A) Transduced THP1 cell lines stably expressing wild type (wt) CD169, mutant (R116A) CD169, ACE2, or both wt CD169 and ACE2. (B) Untransduced primary MDMs from multiple donors showing differential expression of endogenous CD169. After 5–6 days of macrophage differentiation, cells were either unstained or stained with anti-human CD169 antibody, and surface expression analyzed by flow cytometry. (C-D) Representative flow cytometry profiles of primary MDMs transduced with wt CD169 (C) or ACE2 (D) lentiviruses compared to negative (vector only) control. (TIF) [file ppat.1010479.s003.tif]

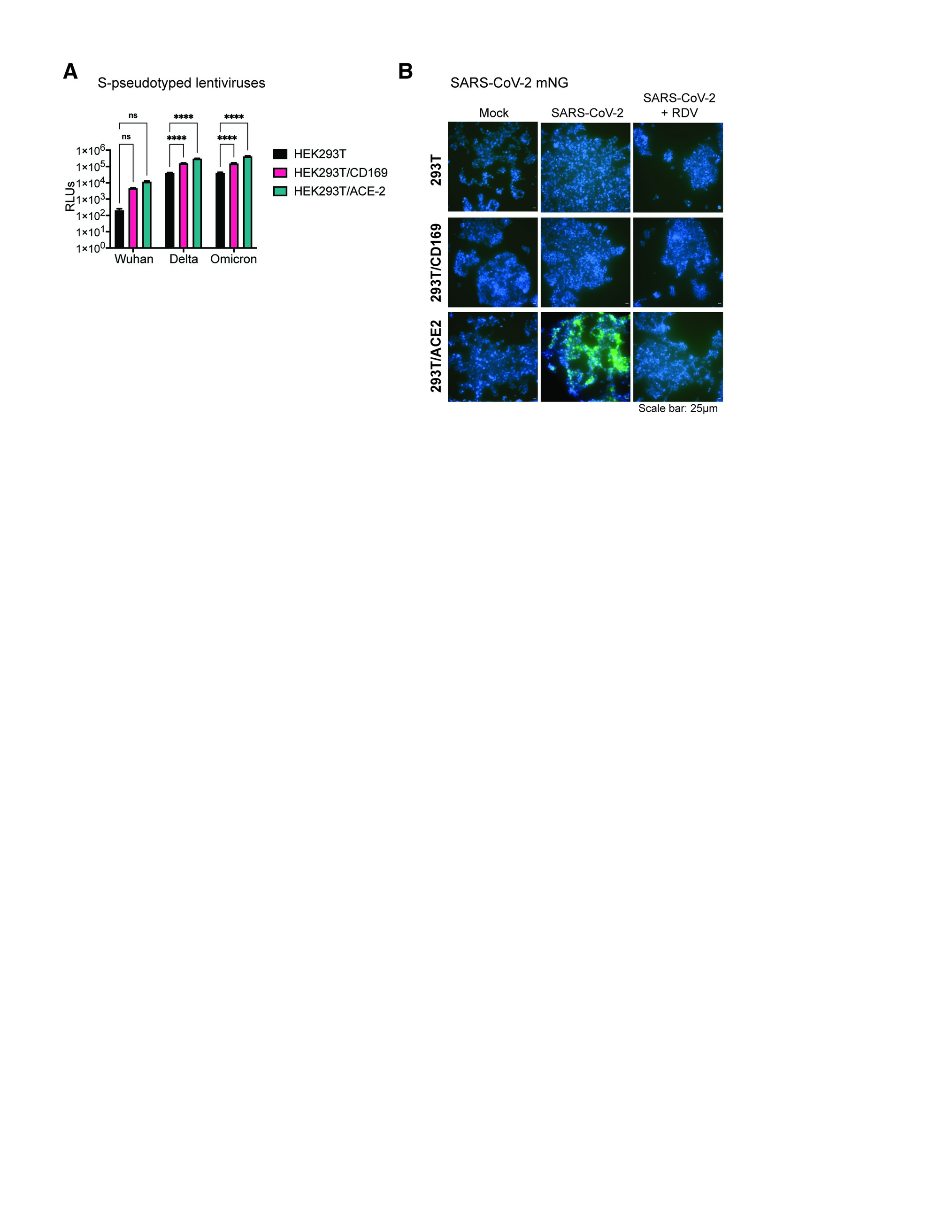

Supplement: S4 Fig — (A) Transduced or untransduced HEK293T cells were infected with either Wuhan, Delta, or Omicron S-pseudotyped lentivirus (20 ng p24Gag) and infection quantified by measuring luciferase expression at 3 dpi. RLUs from each cell line were normalized were normalized to no virus (mock) control. The means ± SEM are shown from 3 independent infections. Significant differences between conditions were determined by one-way ANOVA followed by Tukey’s multiple comparisons test, comparing to untransduced HEK293T cells. P-values: *<0.1; **<0.01; ***<0.001; ****<0.0001. (B) Representative immunofluorescence images (20x) of HEK293T cells overexpressing CD169 or ACE2 compared to untransduced parental line. Cells were treated with DMSO (control) or remdesivir (RDV, 1 μM) for 30 minutes, infected with SARS-CoV-2mNG (MOI = 1) in the absence or presence of RDV, and fixed at 24 hpi followed by staining with DAPI. Images represent 3 independent infections for each cell line. Bar = 25 μm. (TIF) [file ppat.1010479.s004.tif]

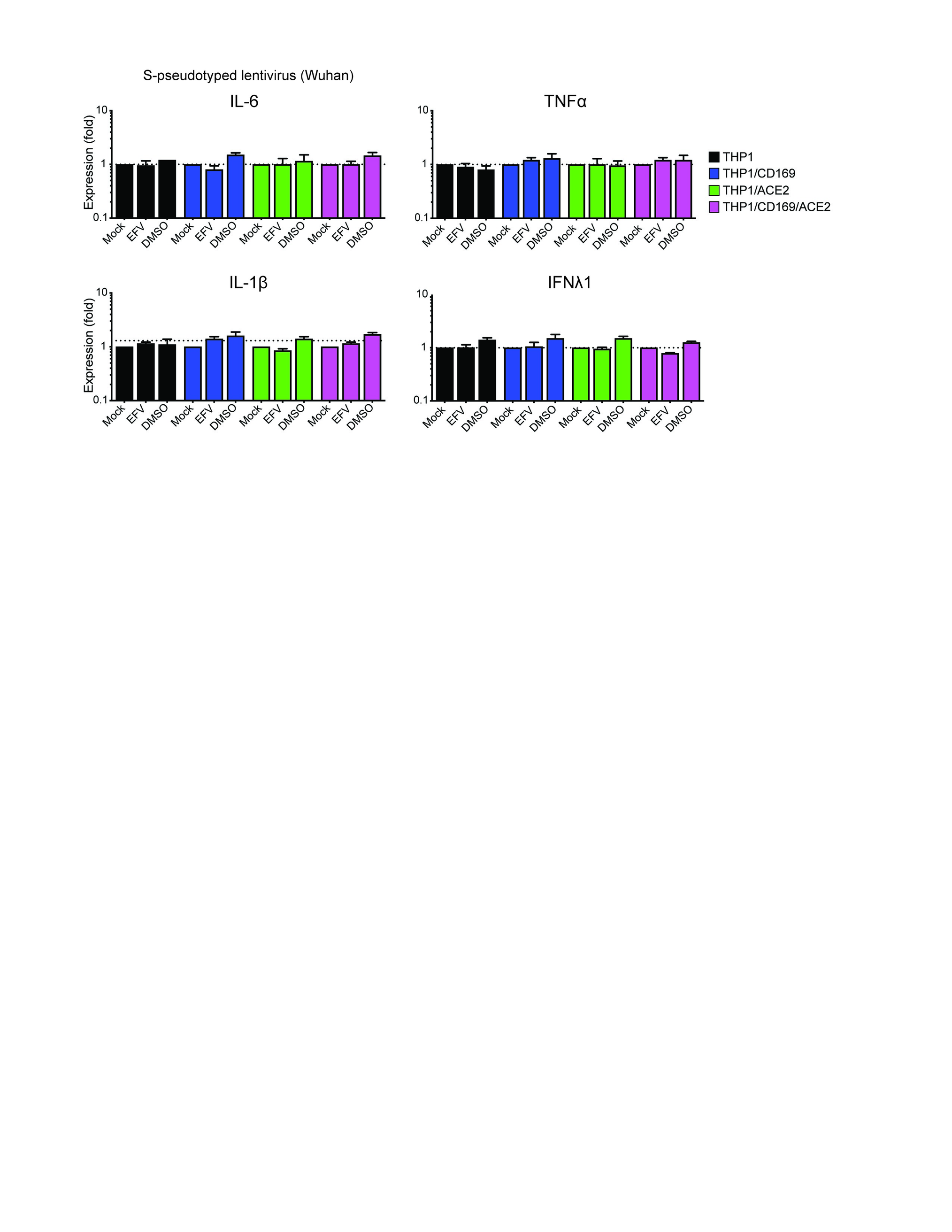

Supplement: S5 Fig — PMA-differentiated THP1 cell lines were infected with SARS-CoV-2 S-pseudotyped lentivirus (20 ng p24Gag) and total RNA was harvested at 2 dpi, followed by qRT-PCR analysis. Fold expression of indicated cytokines normalized to mock (uninfected) condition in each group. Data are representative of at least 3 independent experiments. The means ± SEM from 3 independent experiments are shown. (TIF) [file ppat.1010479.s005.tif]

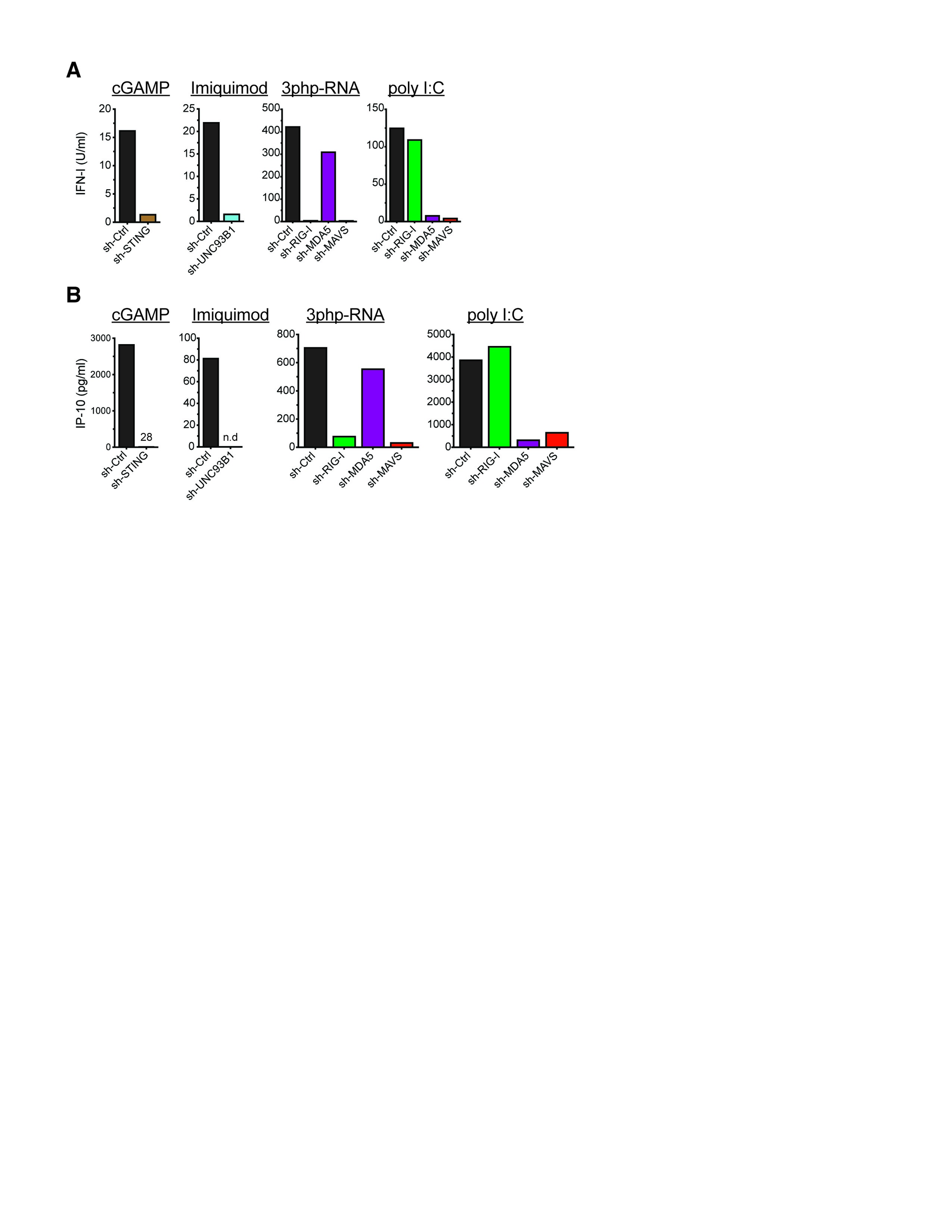

Supplement: S6 Fig — Parental or knockdown (STING, UNC93B1, MAV, RIG-I or MDA5) THP1 cells were stimulated for 24–48 hours with ligands targeting the respective innate immune sensor/adaptor for 24–48 hrs, followed by analysis of culture supernatants for type I IFN production by a bioassay (A), or IP-10 production by ELISA (B). All values normalized to mock (no stimulation) for each cell line. (TIF) [file ppat.1010479.s006.tif]
