# Supplementary material for: CD169-mediated restrictive SARS-CoV-2 infection of macrophages induces pro-inflammatory responses
Source: PLoS Pathog. 2022 Oct 24;18(10):e1010479. doi: 10.1371/journal.ppat.1010479 (PMC9632919; doi:10.1371/journal.ppat.1010479)
Supplement: Attachment — Submitted filename: Jalloh_etal_Response Letter_Final.docx [file ppat.1010479.s008.docx]

We thank the reviewers for their valuable comments and suggested experiments, which are now included in the revised manuscript. To address the reviewers’ concerns, we added **2** new figures (**Figs 5 and 7**), and additional results in **Fig. 2 (panels C & D) and Fig. S4**. Specifically, we included new results that show CD169-mediated, ACE2-independent entry of Delta and Omicron S-pseudotyped lentiviruses (**Fig. 2C-D**).

Using multiple orthogonal approaches, including smFISH and RT-qPCR for viral RNA detection, immunofluorescence staining for viral antigens, and viral titration, we now strengthen our results showing that SARS-CoV-2 entry into primary monocyte-derived macrophages (MDMs) results in the establishment of an abortive infection (**Fig. 5**). We confirmed that cathepsins are required for entry of replication-competent SARS-CoV-2 into MDMs (**Fig. 7A-B**), similar to what we showed before for S-pseudotyped lentiviruses. Furthermore, we now show that exogenous expression of CD169 on non-myeloid HEK293T cells facilitates S-pseudotyped lentivirus entry, but does not support SARS-CoV-2 replication to levels observed in HEK293T/ACE2+ positive cells (**Fig. S4**), suggesting that ACE2-dependent entry is essential for completion of the viral life cycle. Finally, we included new data to show that the induction of inflammatory responses in CD169+ THP1/PMA macrophages and primary MDMs requires initiation of viral RNA synthesis, as remdesivir treatment attenuated IL-6, TNFα, IL-1β, IL-18 and IFNλ expression in both CD169+/THP1/PMA macrophages (**Fig. 6**) and MDMs (**Fig. 7**). Collectively, these new findings, we believe, substantially improve the manuscript and provide strong support of our hypothesis that CD169-mediated SARS-CoV-2 infection of macrophages leads to limited viral RNA synthesis and induction of inflammatory responses in a MAVS-dependent manner.

We include detailed responses to the reviewers’ comments below. Reviewers’ comments are indicated in blue, our responses in black.

**Reviewer #1 Major Comments:**

1. Interpretation/discussion of the data wrt to recently published observations is required (PMID:35385861, 34611663, 33758831, 33277988).”

Both studies (1) (2) suggest a requirement for FcγR-mediated entry of antibody-opsonized SARS-CoV-2 in peripheral blood human monocytes (1) and tissue-resident macrophages in humanized mice with myeloid cell engraftment (2). Both studies suggest that SARS-CoV-2 entry in monocytes/macrophages could be facilitated via antibody-mediated opsonization and FcγRs (CD16 or CD32). Junqueira et al report antibody-opsonized SARS-CoV-2 infection of LPS-activated peripheral blood monocytes, but only 3% of the cells were positive for viral RNA and N protein, though infectious virus production was not observed, suggestive of an abortive infection. Note that in this study, monocytes were shown to not express ACE2, while ACE2 expression on tissue-resident macrophages was not described. In the Sefik et al manuscript, human lung macrophages in the AAV-ACE2 transduced MISTRG humanized mice express ACE2 and are susceptible to SARS-CoV-2 infection in an ACE2 and CD16 (FcγR)-dependent manner. These findings are in contrast to numerous published studies that have reported negligible (to none) ACE2 expression in macrophages in human lung tissues (scRNA seq data from multiple studies (3-6)). Thus, ACE2 expression in lung macrophages in AAV/ACE2/MISTRG humanized mice, a consequence of AAV/AC2 vector transduction might be unique to this animal model. Finally, in contrast to these studies, antibody-dependent enhancement of virus entry in macrophages was not observed in the publication by (7), Rather, virus entry in monocyte-derived macrophages in the Zheng manuscript was dependent on ACE2, but viral infection was restricted at a post-entry step, and virus production was not detected.

We were unable to detect ACE2 expression in primary human MDMs (**Fig. 1**), which is in agreement with other published studies (3-5). Importantly, when ACE2 was exogenously overexpressed in THP1/PMA macrophages or MDMs, virus replication was rescued and resulted in production of infectious virus progeny. It remains possible that the level of ACE2 expression in vitro in MDMs upon lentiviral vector transduction (in our study) is greater than that achieved with AAV transduction in mice (2), or greater than the low levels of endogenous ACE2 expression reported by (7), and that higher levels of ACE2 surface expression can rescue virus replication in macrophages. As suggested by the reviewer, the difference between these recently published studies and our present work is the requirement of antibodies and FcγRs for SARS-CoV-2 entry. We did not perform any infections in the presence of anti-S antibodies or convalescent human plasma. These disparate findings suggest that multiple mechanisms likely contribute to SARS-CoV-2 infection of macrophages

Finally, there is agreement amongst all the previously published studies and our present work that the induction of inflammatory responses in SARS-CoV-2 infected macrophages requires initial viral RNA synthesis as infection with UV-inactivated virus (7), or treatment with remdesivir (1, 2) attenuated induction of cytokine expression. These findings are similar to the results reported by us, in that CD169-mediated virus entry leads to viral infection and induction of pro-inflammatory responses, even though production of infectious virions was restricted. Collectively, these findings suggest existence of diverse independent mechanisms of virus entry into macrophages that contribute to the observed inflammatory phenotype. We hypothesize that CD169 mediated SARS-CoV-2 infection might be especially relevant during the acute phase of virus infection in the lung, especially in the absence of pre-existing humoral responses. These descriptions have been added to the discussion section and reflect the differences as well as the similarities between the various infection paradigms (***lines 504 – 519***).

Please note that both Junqueira (1) and Sefik (2) reports were published after the submission of our manuscript and we cited the preprints in our submission. We have now cited the final peer-reviewed publications in the discussion.

2. In my opinion the use of lentivirus pseudotyped Spike virus was an unfortunate choice for this study given the established role of CD169 in promoting lenti/retroviral infections. As lenti/retrovirus capture by CD169 can occur independent of viral env glycoprotein by binding to sialosides on viral membranes, it is difficult to interpret these data with clarity due to the parallel lentivirus biology at play. A more prudent choice for these experiments would have been SARS-CoV-2 VLPs (PMID:34735219) given that SARS-CoV-2 viral membranes are not plasma membrane-derived like lenti/retroviruses but are from ER-Golgi compartments and may have different set of gangliosides. However parallel data with SARS-CoV-2 live virus somewhat obviates this concern. But the authors should consider acknowledging this limitation.

We agree with the reviewer that gangliosides incorporated within lentivirus particle membranes could contribute to capture by CD169. Previous studies from us and others had indeed discovered that the interaction between the ganglioside GM3 and CD169 was required for HIV-1 capture (8, 9). Interestingly, a recent study suggests that CD169 also recognizes gangliosides incorporated in SARS-CoV-2 particle membranes, and can mediate trans infection of ACE2+ cells (10). However, we show that recombinant S protein binds to CD169 and that binding is abrogated when the sialic acid binding motif in CD169 is mutagenized (R116A mutant, Fig. 2A). Furthermore, to show that entry of the S pseudotyped lentiviruses is mediated by direct S binding, we pre-incubated the lentivirus with antibodies directed against S which blocked infection of MDMs (**Fig. S2**). These results indicate that CD169 interacts with both virus particle-associated gangliosides and sialylated S protein. These descriptions have been added to the manuscript (***lines 488 – 503***).

Reviewer #1 Minor Comments:

1. The authors may want to consider showing that the abortive infection they observe is FcgR-independent by using blocking antibodies.

While FcγRs have recently been shown to contribute to antibody opsonized SARS-CoV-2 entry in macrophages (1), all S-pseudotyped entry studies and SARS-CoV-2 infections in this manuscript were performed in the absence of antibodies. Due to the lack of antibodies in our assays, FcγRs cannot be engaged by virus particles in our infection studies. Our findings suggest an alternative CD169-dependent SARS-CoV-2 entry pathway that establishes an abortive virus replication pathway in macrophages, similar to FcγR mediated virus entry.

2. The induction of RLR-dependent inflammasome activation (IL-18 and IL-b) is interesting. Any ideas how these pathways are linked?

We agree with the reviewer that this is an interesting observation. Numerous studies have linked viral RNA sensing pathways to NLRP3 inflammasome priming and activation. Mechanisms have included MAVS-mediated recruitment of NLRP3 inflammasome at the mitochondrial membrane for optimal inflammasome activation (11), MAVS-dependent NLRP3 oligomerization in Sendai virus infected cells (12), RIG-I and MDA5- mediated sensing of influenza A viral RNA intermediates and MAVS-dependent activation of the inflammasome components, IL-1β, IL-18 and NLRP3 (13). Our findings suggest that RIG-I, MDA5 and MAVS are required for sensing SARS-CoV-2 replication intermediates and that sensing of viral RNA replication intermediates primes inflammatory responses. However, a requirement for MAVS-mediated NLRP3 inflammasome activation was not directly tested in our studies so that we cannot comment on a specific mechanism. To make this clearer to the readers, we have added a sentence to the discussion section which now reads as follows: “*Besides MAVS-mediated upregulation of IL-1β and IL-18 expression, viral RNA sensing pathways have also been linked to NLRP3 inflammasome priming and activation via MAVS-mediated recruitment of NLRP3 inflammasome at the mitochondrial membrane for optimal inflammasome activation (88). Thus, SARS-CoV-2 infected and primed CD169^+^ macrophages might uniquely contribute to the inflammasome activation upon delivery of secondary activation signals and perpetuate the hyper-inflammatory phenotype*.” (***lines 597 – 603***).

3. Figure 5A and G: The induction of IL-1 beta and IL-18 in THP-1 CD169 but not in ACE2 expressing cells is also interesting and reveals activation CD169-entry dependent activation of inflammasomes. Also I am a little surprised by the expression of proinflammatory cytokines and IFNlambda despite remdesivir treatment in MDMs (Figure 5G). Although significantly lower than without RDV, expression levels are still 10-30 fold above the background suggesting a virus replication-independent sensing.

We are also intrigued by the finding of selective IL-1β and IL-18 induction upon CD169-mediated SARS-CoV-2 entry, as opposed to that observed in ACE2-infected macrophages. Since a recent study reported that enforced NLRP3 inflammasome activation can suppress SARS-CoV-2 replication in macrophages (2), it is intriguing to speculate that SARS-CoV-2 encodes antagonisms of the NLRP3 inflammasome activation pathway, and that lack of establishment of a productive infection in ACE2-deficient CD169+ macrophages prevents suppression of inflammasome activation. The reviewer is correct in suggesting that while remdesivir treatment substantially decreases inflammatory cytokine expression, the suppression was not complete. It remains formally possible that binding to TLR2 or other C-type lectin receptors on macrophages can partially induce inflammatory cytokine gene expression. Though it should be noted that in our infection model, viral RNA (replication intermediates) sensing contributed predominantly to inflammatory responses. To highlight this we edited the discussion to now read as follows: “*Critically, treatment with E64D (cathepsin inhibitor) or RDV not only blocked de novo viral RNA expression but also significantly reduced pro-inflammatory cytokine expression in non-productively infected CD169^+^ macrophages, suggesting that viral RNA (replication intermediates) sensing contributed predominantly to inflammatory responses (****Figs. 6*** *and* ***7****). The lack of complete suppression of proinflammatory cytokines upon RDV treatment suggests that virus binding to TLR2 or capture by C-type lectin receptors can also partially induce inflammatory cytokine gene expression”* (***lines 557 – 563***).

4. Figure 4C: With regard to point 4 and CD169-dependent inflammasome activation, are the THP-1/CD169 cells dying (pyroptosis) resulting in reduced N expression ? Were the expression measured wrt to a house keeping gene ? This would be required to interpret the data. (LPS was not used, and hence no activation of the inflammasome)

We apologize if the PCR experimental conditions were not explicitly described. A detailed description of the PCR conditions has been included in the revised version of the manuscript. All RT-qPCR analysis included quantification of housekeeping gene (GAPDH) and the expression of cytokines was normalized to GAPDH expression (Δ^CTT^) (***lines 924 – 927***). Moreover, analysis of level of GAPDH in RNA samples revealed no difference between mock or infected cells, suggesting no cytopathic effect of partial virus replication in THP1/CD169 cells

**Reviewer #2 Major Comments:**

1. In the experiments shown in figure 2, the investigators show that CD169 mediates part of this infection in THP1 monocytic cell lines. Did the investigators express CD169 in 293T cells or other cells that do not express ACE2 to examine if CD169 alone can mediate infection in these cells? This would be preferable to do in a cell line that is highly sensitive to infection and not macrophages.

This is an excellent suggestion. In response to the reviewer’s comment, we established HEK293T cells constitutively expressing CD169 or ACE2 and infected them with S-pseudotyped lentiviruses and infectious SARS-CoV-2. Similar to the results obtained with THP1/CD169 cells, there was robust enhancement of S-pseudotyped lentivirus infection of CD169 expressing HEK293T cells (**Fig. S4**; 10-fold enhancement) that did not result in viral replication, suggesting that CD169 is a viral entry factor, but CD169-mediated virus entry does not lead to productive infection. This was different in HEK293T/ACE2 expressing cells that showed robust SARS-CoV-2 infection, reflecting our data obtained with transduced THP1/PMA cells and MDMs. These findings are described in a new supplementary figure (**Fig. S4**).

2. Have the authors validated the role of CD169 in modulating infection in vivo? Do CD169 KO mice have differences in SARS-CoV-2-mediated lung pathology? Conversely, and getting at the same question, can inhibition of CD169 via 7D2 in mice dampen SARS-CoV-2 mediated inflammation in infected mice?

The role of CD169 in SARS-CoV-2 infection in vivo has not been determined. These studies will require infection of CD169 knock-out mice to conclusively determine the role of CD169 in viral pathogenesis. While these are important experiments, we believe such studies are beyond the scope of the present manuscript.

3. How were the live SARS-CoV-2 virus infections done in the THP1/ACE2 and CD169-expressing cells? And in MDM expressing ACE2? Is there a wash step to remove unbound virus before quantifying infectious viral titers? Why is only the ACE2-expressing MDM mediating virus infection? Is there a possibility that SARS-CoV-2 live virus remains attached to ACE2 but not the CD169-expressing MDMs (Figure 3F) and this is driving the replication observed? This should be clarified.

We apologize for this oversight. Experimental conditions are now clearly described in the methods section (***lines 852 – 865***). Following virus incubation for 1h, all cells were vigorously washed to remove unbound viral particles. Cells were then cultured in fresh media ± inhibitors for indicated time. We observed similar levels of recombinant S binding to THP1/CD169 or THP1/ACE2 cells (**Fig. 2A**). And there was no difference in the amount of viral gRNA (N transcripts) at 2h post infection in virus infected THP1/CD169+ and THP1/ACE2+ macrophages. Both lines of experiments suggest that virus capture/binding and entry is equivalent when S binds to CD169 or ACE2, but the subsequent steps in the viral replication cycle are different, leading to abortive infection after CD169-mediated entry and productive infection after ACE2-mediated entry.

Reviewer #2 Minor Comments:

4. How about different SARS-CoV-2 variant spikes? Are pseudotypes expressing delta or omicron spikes more infectious compared to the Wuhan-1 spike via the CD169 entry mechanism? Especially given that omicron may prefer the endocytic pathway, it would be particularly interesting to evaluate pseudotyped spikes expressing B.A.1 or other omicron sublineages.

Following the reviewer’s suggestion, we have repeated the pseudotyped lentivector infection studies with omicron and delta S pseudotyped lentiviral vectors in THP1/CD169 macrophages (**Fig. 2-D**) and HEK293T/CD169 cells (**Fig. S4A**). In both cell types, CD169 overexpression dramatically enhanced both delta and omicron S-pseudotyped lentivirus infection equivalently, suggesting that entry of omicron S pseudotyped lentiviruses is not selectively enhanced by CD169.

5. Can the authors elaborate on the functional relevance of CD169 expression macrophages from COVID-infected people? While their CD169 data in THP1 cells is interesting, what does this mean in the broader context?

CD169 is constitutively expressed on alveolar macrophages and is induced on monocytes and monocyte-derived macrophages under inflammatory conditions. RNAseq analysis and immunohistochemistry of lung biopsies from COVID patients demonstrates upregulation of CD169 expression in alveolar macrophages and interstitial macrophages (14-17). These expression analyses suggest that induction of CD169 on tissue-resident macrophages might enhance SARS-CoV-2 entry and infection establishment in macrophages that could promote the hyperinflammatory phenotype and contribute to COVID19 disease severity. This information is now included in introduction of the edited manuscript (***lines 113 – 121***).

6. In the macrophage phagocytosis experiments that examined pseudotypes expressing SARS-CoV-2 spike, did the authors also examine a negative control NL63 spike to examine the background infectivity of these cells via the endocytic pathway. This control should also be shown in Figure 1. Currently, it is unclear what the background infection is with this system.

We are unclear about the reviewer’s proposed experiment. NL63 S also binds to ACE2 for entry and as such might not serve as the optimal control for these studies. We demonstrate that pre-treatment with either anti-CD169 neutralizing antibody (7D2, **Fig. 2F**) or anti- S monoclonal antibodies (**Fig. S2**), significantly attenuated S-pseudotyped lentivirus entry, suggesting the specificity of this interaction. Furthermore, treatment with E64D (cathepsin inhibitor) potently blocked both S-pseudotyped lentivirus entry (**Fig. 1**) and also replication competent SARS-CoV-2 replication (**Fig. 7**) in MDMs in newly included experiments, suggesting that endosomal entry is the predominant mode of virus entry in MDMs.

**Reviewer #3 Major Comments:**

1. The findings in Figure 1 should be substantiated with actual SARS-CoV-2 infection. Latter studies were conducted with live virus infections using modified THP1 cells, but not primary MDMs. The studies in Figure 3 primarily featured infection data in primary MDMs transduced to over-express CD169 or ACE2, but not unmodified MDMs.

We think the reviewer might have missed the data that includes SARS-CoV-2 infections of primary MDMs (*Figs. 3G, 4G-H, and 5G* **in the prior submission**) and apologize for the confusing presentation of our data. To highlight the importance of experiments using primary MDMs, we revised and restructured the manuscript to highlight our findings in primary MDMs and included them as new figures (**Figs. 5 and 7**). Similar to the results with PMA-differentiated THP1/CD169 macrophages, SARS-CoV-2 infection of MDMs resulted in abortive virus infection (**revised Fig. 5**). No infectious progeny virions were detected in supernatants of untransduced (parental) MDMs infected with SARS-CoV-2 (**Fig. 5F**), and CD169 overexpression did not rescue SARS-CoV-2 replication in MDMs. In contrast, ACE2 overexpression in MDMs was sufficient to restore robust levels of virus replication and infectious virus production (**Fig. 5F**). Importantly, restricted virus replication in untransduced MDMs (as determined by detection of viral gRNA and sgRNAs by RT-qPCR), was the trigger for inflammatory cytokine expression as E64D (cathepsin inhibitor, **Fig 7**) or remdesivir pre-treatment (**Fig. 7**), attenuated IL-1β, IL-6, TNFα IL-29 and IP-10 expression, suggesting that expression of viral replication intermediates in MDMs induces inflammatory cytokine expression.

2. Is live virus infection of macrophages require TMPRSS2?

We did not observe TMPRSS2 expression in MDMs (**Fig. S1**) yet expression of ACE2 resulted in SARS-CoV-2 infection (**Fig. 3** and **Fig. 5**). Upon the reviewer’s request and to further investigate the potential role of TMPRSS2 in macrophages, we infected MDMs with SARS-CoV-2 in the presence or absence of E64D (cathepsin inhibitor) or camostat (TMPRSS2 inhibitor), and observed that only E64D blocks initiation of viral RNA synthesis, suggesting that CD169-mediates endosomal entry of SARS-CoV-2 in macrophages, and does not require TMPRSS2. These new data have been included in the manuscript (**Fig. 7A** and **B**).

3. What are the sialylated motifs recognized by CD169 on SARS-CoV-2 spike protein? The findings in Figure 2 suggest that this is the case, but not conclusive data were presented. (cite references with sialylation, and glycosylation)

Our data suggest that terminal sialic acids on the S protein are recognized by CD169 because S did not bind to the CD169/R116A mutant (**Fig. 2**). This mutant is deficient in sialic acid binding. SARS-CoV-2 S is extensively glycosylated with some of the complex glycans having terminal sialic acid residues (18, 19). While it would be informative to map the exact CD169 binding sites on S, it is beyond the scope of this manuscript.

4. How does the over-expression of CD169 compare to the physiologically relevant expression of CD169 on lung-resident macrophages (alveolar; interstitial or MDMs)? (cited: CD169 monocytes on patients)

CD169 is constitutively expressed on alveolar macrophages and is induced on monocytes and monocyte-derived macrophages under inflammatory conditions. RNAseq and immunohistochemistry analysis of lung tissues from COVID patients demonstrated upregulation of CD169 expression in alveolar macrophages and interstitial macrophages (15, 17, 20). These data suggest that induction of CD169 might enhance SARS-CoV-2 infection of macrophages that could promote the hyperinflammatory phenotype observed in severe COVID19. This information has been added to the manuscript (***lines 113 – 121***).

5. The authors should include UV-inactivated virus to show the robustness of the RNA FISH staining in Figure 4A. Further, how do these findings compare with unmodified\untransduced primary MDMs infected with SARS-CoV-2?

We believe that we have included sufficient data to demonstrate specificity and robustness of the FISH staining, with complete lack of FISH signals in mock-infected cells, in untransduced infected THP1 cells, and at early time points post infection (1-2 hpi) (**Fig. 4A**), indicating that our probes do not generate any false positive signals. smFISH is a well-established and popular technique with 1800 articles citing the first smFISH paper (21). The specificity in smFISH stems from the fact that many probes bind to the same target molecule for it to become visible.  smFISH has been used for many applications, including to show distinct expression of heterologous mRNAs and expression of inducible genes. The robustness of the method has been tested by including probe-based controls, such as sense or irrelevant probe sets, which have not generated any signals.  Additional information regarding the specificity of signals in smFISH is provided in (22).  In light of this, we think that infection studies with UV-inactivated virus are not necessary to demonstrate specificity of our assay.

With regards to MDMs, we have now included data to show smFISH staining of MDMs infected with SARS-CoV-2 (**Fig. 5A**). Similar to the findings with THP1/PMA macrophages, only ACE2 expression robustly increases perinuclear viral RNA signals, while expression of CD169 alone results in diffuse cytoplasmic staining with isolated puncta. These results with MDMs validate our findings in THP1/PMA macrophages.

6. How are the authors able to detect negative-sense SARS-CoV-2 RNA? No evidence is provided by the authors beyond a citation that negative strand vRNA is detected in their assay.

We apologize for this oversight. The RT step utilized gene specific primers that only bind to the negative strand viral RNA, as previously demonstrated by (23). Reverse-transcribed products were used as templates for amplification using gene specific primers. For RT reaction control, we used plus strand primers that failed to generate PCR products. As negative control for amplification, we used primers targeting sequences outside the RT products. Both controls failed to generate any amplification signal by SYBR Green. The following information has been added to the methods section (***lines 912 – 924***):

“*For detection of negative-strand viral RNA, the reverse transcription primer 5’-* ***ACAGCACCCTAGCTTGGTAG****CCGAACAACTGGACTTTATTGA -3’ was used, and it consists of two parts. The first sequence is labeled in bold and underlined, and serves as an internal amplification control which is not present in the viral RNAs. The second part targets ORF1ab gene from 647 to 688bp of SARS-CoV-2 (isolate USA_WA1/2020). For amplification, the forward primer, 5’- AGGTGTCTGCAATTCATAGC-3’ (743-762bp), and the reverse primer, 5’-* ***ACAGCACCCTAGCTTGGTAG*** *-3’ were used. As a negative control for amplification, the primer, 5’-CCCTGATGGCTACCCTCTT-3’ (583-602bp) was used. Reverse transcription was performed with Superscript III 1st Strand cDNA Synthesis kit (Invitrogen), and PCR was carried out using Maxima SYBR Green (Thermo Scientific).”*

7. Figure 5- The authors should include UV-inactivated controls and dose titration studies to demonstrate that the inflammation observed in primary MDMs is indeed due to virus infection and not an artifact in the media within the virus inoculum.

We are confident with the conclusion that inflammatory cytokine expression in MDMs is due to sensing of viral replication intermediates, since infections in the presence of remdesivir and E64D (new results included in the revised manuscript) attenuated inflammatory responses (**Fig. 7C**). We used purified (ultracentrifugation) replication competent viruses for these experiments. Robust decrease in inflammatory cytokine expression upon inhibition of virus entry (E64D) or virus replication (remdesivir) strongly suggests that virus exposure is not sufficient, but rather initiation of virus replication is required for induction of inflammatory responses in SARS-CoV-2 infected MDMs.

8. The authors have not adequately demonstrated that innate immune activation requires de novo viral RNA synthesis.

We respectfully disagree with the reviewer on this point. Please see response to comment #7 of this reviewer and the results in **Fig. 7**, which conclusively demonstrate that remdesivir treatment blocks induction of inflammatory cytokines and that S-pseudotyped lentivirus infection does not induce any pro-inflammatory cytokines (**Fig. S5**).

9. What is the in vivo role of MAVS in macrophages during SARS-CoV-2 infection? (MAVS k/o mice data: is there a publication?)

To our knowledge, no reports have been published to define the role of MAVS in SARS-CoV-2 infection in animal models, as MAVS k/o mice have not been utilized in virus challenge experiments.

**References**

1.	Junqueira C, Crespo A, Ranjbar S, de Lacerda LB, Lewandrowski M, Ingber J, et al. FcgammaR-mediated SARS-CoV-2 infection of monocytes activates inflammation. *Nature.* 2022;606(7914):576-84.

2.	Sefik E, Qu R, Junqueira C, Kaffe E, Mirza H, Zhao J, et al. Inflammasome activation in infected macrophages drives COVID-19 pathology. *Nature.* 2022;606(7914):585-93.

3.	Bost P, Giladi A, Liu Y, Bendjelal Y, Xu G, David E, et al. Host-Viral Infection Maps Reveal Signatures of Severe COVID-19 Patients. *Cell.* 2020;181(7):1475-88 e12.

4.	Hikmet F, Mear L, Edvinsson A, Micke P, Uhlen M, and Lindskog C. The protein expression profile of ACE2 in human tissues. *Mol Syst Biol.* 2020;16(7):e9610.

5.	Lu Q, Liu J, Zhao S, Gomez Castro MF, Laurent-Rolle M, Dong J, et al. SARS-CoV-2 exacerbates proinflammatory responses in myeloid cells through C-type lectin receptors and Tweety family member 2. *Immunity.* 2021;54(6):1304-19 e9.

6.	Tang L, Yin Z, Hu Y, and Mei H. Controlling Cytokine Storm Is Vital in COVID-19. *Front Immunol.* 2020;11:570993.

7.	Zheng J, Wang Y, Li K, Meyerholz DK, Allamargot C, and Perlman S. Severe Acute Respiratory Syndrome Coronavirus 2-Induced Immune Activation and Death of Monocyte-Derived Human Macrophages and Dendritic Cells. *J Infect Dis.* 2021;223(5):785-95.

8.	Puryear WB, Akiyama H, Geer SD, Ramirez NP, Yu X, Reinhard BM, et al. Interferon-inducible mechanism of dendritic cell-mediated HIV-1 dissemination is dependent on Siglec-1/CD169. *PLoS Pathog.* 2013;9(4):e1003291.

9.	Puryear WB, Yu X, Ramirez NP, Reinhard BM, and Gummuluru S. HIV-1 incorporation of host-cell-derived glycosphingolipid GM3 allows for capture by mature dendritic cells. *Proc Natl Acad Sci U S A.* 2012;109(19):7475-80.

10.	Perez-Zsolt D, Munoz-Basagoiti J, Rodon J, Elosua-Bayes M, Raich-Regue D, Risco C, et al. SARS-CoV-2 interaction with Siglec-1 mediates trans-infection by dendritic cells. *Cell Mol Immunol.* 2021;18(12):2676-8.

11.	Subramanian N, Natarajan K, Clatworthy MR, Wang Z, and Germain RN. The adaptor MAVS promotes NLRP3 mitochondrial localization and inflammasome activation. *Cell.* 2013;153(2):348-61.

12.	Park S, Juliana C, Hong S, Datta P, Hwang I, Fernandes-Alnemri T, et al. The mitochondrial antiviral protein MAVS associates with NLRP3 and regulates its inflammasome activity. *J Immunol.* 2013;191(8):4358-66.

13.	Pothlichet J, Meunier I, Davis BK, Ting JP, Skamene E, von Messling V, et al. Type I IFN triggers RIG-I/TLR3/NLRP3-dependent inflammasome activation in influenza A virus infected cells. *PLoS Pathog.* 2013;9(4):e1003256.

14.	Delorey TM, Ziegler CGK, Heimberg G, Normand R, Yang Y, Segerstolpe A, et al. COVID-19 tissue atlases reveal SARS-CoV-2 pathology and cellular targets. *Nature.* 2021;595(7865):107-13.

15.	Doehn JM, Tabeling C, Biesen R, Saccomanno J, Madlung E, Pappe E, et al. CD169/SIGLEC1 is expressed on circulating monocytes in COVID-19 and expression levels are associated with disease severity. *Infection.* 2021;49(4):757-62.

16.	Grant RA, Morales-Nebreda L, Markov NS, Swaminathan S, Querrey M, Guzman ER, et al. Circuits between infected macrophages and T cells in SARS-CoV-2 pneumonia. *Nature.* 2021;590(7847):635-41.

17.	Lee JS, and Shin EC. The type I interferon response in COVID-19: implications for treatment. *Nat Rev Immunol.* 2020;20(10):585-6.

18.	Watanabe Y, Allen JD, Wrapp D, McLellan JS, and Crispin M. Site-specific glycan analysis of the SARS-CoV-2 spike. *Science.* 2020;369(6501):330-3.

19.	Yang Q, Hughes TA, Kelkar A, Yu X, Cheng K, Park S, et al. Inhibition of SARS-CoV-2 viral entry upon blocking N- and O-glycan elaboration. *Elife.* 2020;9.

20.	Zhou Z, Ren L, Zhang L, Zhong J, Xiao Y, Jia Z, et al. Heightened Innate Immune Responses in the Respiratory Tract of COVID-19 Patients. *Cell Host Microbe.* 2020;27(6):883-90 e2.

21.	Raj A, van den Bogaard P, Rifkin SA, van Oudenaarden A, and Tyagi S. Imaging individual mRNA molecules using multiple singly labeled probes. *Nat Methods.* 2008;5(10):877-9.

22.	Batish M, van den Bogaard P, Kramer FR, and Tyagi S. Neuronal mRNAs travel singly into dendrites. *Proc Natl Acad Sci U S A.* 2012;109(12):4645-50.

23.	Liao M, Wu J, Dai M, Li H, Yan N, Yuan R, et al. Rapid detection of SARS-CoV-2, replicating or non-replicating, using RT-PCR. *Int J Infect Dis.* 2021;104:471-3.
